# Hybrid Lipid/Clay Carrier Systems Containing Annatto Oil for Topical Formulations

**DOI:** 10.3390/pharmaceutics14051067

**Published:** 2022-05-17

**Authors:** Raquel de Melo Barbosa, Aliana Monteiro Leite, Fátima García-Villén, Rita Sánchez-Espejo, Pilar Cerezo, César Viseras, Angela Faccendini, Giuseppina Sandri, Fernanda Nervo Raffin, Túlio Flávio Accioly de Lima e Moura

**Affiliations:** 1 Laboratory of Drug Development, Department of Pharmacy, Federal University of Rio Grande do Norte, Natal 59012-570, Brazil; alianamonteiro@hotmail.com (A.M.L.); tulio.moura@ufrn.br (T.F.A.d.L.e.M.); 2 Department of Pharmacy and Pharmaceutical Technology, School of Pharmacy, University of Granada, Campus of Cartuja s/n, 18071 Granada, Spain; fatima.garciav@ehu.eus (F.G.-V.); ext.risanchez@ugr.es (R.S.-E.); mcerezo@ugr.es (P.C.); cviseras@ugr.es (C.V.); 3 Andalusian Institute of Earth Sciences, CSIC-University of Granada, Av. de las Palmeras 4, Armilla, 18100 Granada, Spain; 4 Department of Drug Sciences, University of Pavia, Viale Taramelli 12, 27100 Pavia, Italy; angela.faccendini@gmail.com (A.F.); g.sandri@unipv.it (G.S.)

**Keywords:** hybrid system, topical formulations, colloidal suspension, *Bixa orellana* L.

## Abstract

Nanocomposites formed by clay and lipid carriers (NLCs) show a high potential for providing controlled release and specific delivery of bioactive molecules and have recently gained attention in the pharmaceutical sector due to their ability to transport hydrophilic and hydrophobic drugs. Recent studies have recognized the biological activity of the oil of *Bixa orellana* L. (AO) with regards to its healing, antioxidant, antibacterial, and anti-leishmanial properties. Therefore, the purpose of this study is the preparation and characterization of hybrid systems based on lipid nanocarriers and laponite for the delivery of AO. NLCs were prepared by the fusion-emulsification method, using cetyl palmitate (CP) or myristyl myristate (MM), AO, and Poloxamer 188. The morphology, hydrodynamic diameters, zeta potential (ZP), polydispersity index (PDI), thermal analysis, X-ray diffraction analysis (XRD), viscosity behavior, and cytotoxicity testing of the hybrid systems were performed. The thermal study and X-ray diffraction analyses (XRD) revealed polymorphic structural changes compatible with the amorphization of the material. Rheological assays highlighted a typical pseudoplastic behavior in all systems (MM and CP with LAP). The hybrid systems’ morphology, size diameters, and PDIs were similar, preset spherical and monodisperse structures (≈200 nm; <0.3), without significant change up to sixty days. The ZP values differed from each other, becoming higher with increasing AO concentration. XEDS spectra and elemental X-ray maps show peaks of lipids (organic components, C and O) and inorganic components O, Mg, and Si. All samples showed cell viability above 60%. The results indicated a stable, biocompatible hybrid system that can be an alternative for topical application.

## 1. Introduction

Conceptually, nanocomposite systems are formed by two or more materials with different characteristics that seek to conjugate their properties to obtain a superior material [1]. Nanocomposites have dispersed particles where there is at least one nanomaterial, and often there are substantial interactions between the particles formed: for example, between polymer and clay [2], or lipid and clay [3]. These systems attract considerable interest due to their wide range of advantageous properties compared to pure pristine materials; increased mechanical strength, thixotropy, reduction in gas permeability, and higher heat resistance [1,4].

Nanocomposites formed by clay and biomaterials (proteins, lipids, polysaccharides, etc.) have a high potential for providing predictable, accurate, and reproducible patterns of controlled release and enabling specific delivery of bioactive molecules [5,6,7]. Natural, modified, and synthetic clays can be considered for the development of nanocomposites [8,9]. These include hectorite, montmorillonite, saponite and other smectites (natural clays), organo-smectites (modified clays), fluorohectorite, fluoromic, and laponite (synthetic clays), due to their distinct advantages: they are versatile (due to their wide range of mechanical, chemical, and physical properties), generally considered inert, and are available at reasonable cost [5].

Laponite RD (LAP) type clay nanoparticles are biocompatible and disk-like [10], and are widely used as rheology modifiers in various technological applications. In addition, they may also form hydrogels due to interactions between their termini, adopting a ‘house of cards’ type structure. Hydrogels have become a subject at the heart of much research because of their similarity to natural tissue and/or as a reservoir for localized and sustained release of drugs [11,12].

Studies have shown the hybridization of different clays with polymers or surfactants by strong secondary interactions of the mineral compounds with their guests. The interaction between these compounds, including the attachment of drugs [13], can promote satisfactory characteristics such as cell adhesion [14], proliferation and differentiation of cells [15], and cellular uptake [16]. Therefore, these hybrid nanocomposites have been suggested for use in chronic wound treatment, with the expectation of increasing wound healing effectiveness.

Lipid nanoparticles are based on ‘generally recognized as safe’ (GRAS) materials and are characterized by their excellent stability, biocompatibility, and low dermal toxicity [17]. In addition, they are low cost, easy to prepare, and present high loading efficiency for hydrophobic drugs. Lipid nanoparticles comprise solid lipid nanoparticles (SLNs), nanostructured lipid carriers (NLCs), and lipid drug conjugates (LNCs) [18]. SLNs are lipid carriers, consisting of one or more solid lipids dispersed in the presence of a colloidal stabilizer. NLCs exist as ‘second generation’ lipid nanoparticles. Different from SLNs, their lipid composition is a combination of solid and liquid lipids, which gives elevated protection to the loaded drugs and less drug leakage from their structures [19,20,21,22]. LNCs are nano-vesicular systems that exhibit a core-shell appearance. 

It is noteworthy that the release kinetics of the encapsulated drug can be controlled by adjusting the composition of the lipid nanoparticles. It is clear that the morphological control of the particles—particularly the membrane thickness—is crucial for the definition of the drug release profile. Regarding the topical application of lipid nanoparticles (SLNs and NLCs), Müller and collaborators in 2011 described the occlusive effect of the particles on the stratum corneum, due to the development of a lipid film formed by their deposition after application on the skin. Adherence is due to hydrophobic interactions occurring between the lipid components of the skin and the formulation, promoting an increase in both the degree of hydration, reinforcement, and repair of the skin, as well as greater penetration of drugs in the deeper layers of the cutaneous tissue [23,24,25].

Herbal medicines can be a low-cost and accessible alternative in wound healing management. *Bixa orellana* L. is a plant of the family Bixaceae and genus Bixa, commonly known as urucum or annatto. In addition to its applications in mainstream medicine, several scientific studies have sought to elucidate the effectiveness of *Bixa orellana* L. in different disease states. Medina-Flores and collaborators in 2016 confirmed the antibacterial activity of the anatto methanolic extract against *Streptococcus mutans* and *S. sanguinis* [26,27]. Additionally, Fleischer and colleagues, 2003, demonstrated the antifungal activity of the ethanol extract from leaves and seeds against *Candida albicans* [27]. The activity of the essential oil of *Bixa orellana* L. seeds against *Leishmania amazonensis* was demonstrated by Monzote and collaborators in 2014. Several studies are addressing the potential of this plant for diversified application, including its use for the treatment of the so-called ‘neglected’ diseases and for tissue regeneration [28].

According to Vilar et al., 2014 [29], the fatty acids extracted from *Bixa orellana* L. seeds (AO), exhibited pro-inflammatory action, a characteristic that can accelerate the healing process and suggests a use for tissue repair, especially in the treatment of wounds where there is a risk of infection. According to Frega et al., 1998, the oily fraction of *Bixa orellana* L. contains tocol methyl [3,4-dihydro-2-methyl-(4′,8′,12′-trimethyl-tridecyl)-2H-1-benzopyran-6-ol] derivatives (~90% delta-tocotrienol, 10% gamma-tocotrienol) in saturated (tocopherols) and unsaturated (tocoenols) forms. Tocotrienols (vitamin E) are natural fat-soluble antioxidant compounds with recognized biological importance [30,31]. Their effects are being studied in infected wounds, different types of cancer, bone resorption, diabetes, and cardiovascular and neurological diseases. In addition to tocotrienols, identified in the AO seeds were: alkaloids, flavonoids, other carotenoids, gallic acid, orelin, di, mono, and sesquiterpenes, and palmitic and linoleic acids, with bixin being the main component identified in annatto oil, which corresponds to about 80% of the compounds present in AO [31].

One of the enormous challenges in clinical medicine is the rapid and efficient treatment of complex and chronic wounds. Unfortunately, this is a subject still much discussed and is considered a serious world health problem. Chronic wound states such as diabetic foot, venous leg ulcers, and pressure ulcers all have common features (prolonged or excessive inflammation, persistent infection, formation of drug-resistant microbial biofilms, and an inability of the dermal and/or epidermal cells to respond to repair stimuli) [4,32,33].

At present, most of the wound dressings available are unable to solve the problems related to chronic wounds, like bacterial resistance, protein adsorption, and increased levels of exudates. The biological properties of therapeutic dressings are related not only to the drugs used, but also to the presence of polymers or clay or lipid nanoparticles that can improve tissue repair, reducing the local inflammatory response more efficiently [4,33,34].

This work thus includes the preparation and characterization of hybrid systems formed with nanoparticulate lipid systems and clays for the delivery of AO. Emphasis will be placed on the study of lipid nanoparticles, clays, and nano-hybrid systems with extensive characterization and optimization of the systems.

## 2. Materials and Methods

### 2.1. Materials

Crodamol™ MM (Myristyl Myristate, MM_bulk_), Crodamol™ CP (Cetyl Palmitate, CP_bulk_), and Synperonic™ PE (poloxamer 188, PL_bulk_) were received as free samples from Croda (Campinas, Brazil). The oily fraction, annatto oil (AO), from *Bixa orellana* L. seeds was supplied by Chr. Hansen (São Paulo, Brazil), and Laponite^®^ RD (LAP_bulk_) by BYK Additives & Instruments (Wesel, Germany). Fibroblasts (normal human dermal fibroblasts (nHDF) from juvenile foreskin, PromoCell, WVR, Milan, Italy). All other chemicals and solvents were of analytical grade. Ultrapure water was obtained from a Milli Q^®^ apparatus (Millipore^®^, Burlington, MA, USA).

### 2.2. Methods

#### 2.2.1. Preparation of Nanoparticles

Nanoparticles were prepared by ultrasonication with different components (Table 1) following a procedure performed by Barbosa et al., (2018 and 2013) [35,36,37] with some modifications. MM_bulk_ or CP_bulk_, a solid lipid at 25 °C, was heated to 60 °C with the liquid lipid and active compound, AO. An aqueous solution of PL*_bulk_* was also heated to the same temperature as the oily phase. The aqueous phase was then mixed with the oily phase for three minutes at 10,000 rpm using Ultra-turrax T18 (IKA Work, Staufen, Germany). This pre-emulsion was sequentially homogenized for fifteen minutes (30 s on, 30 s off) at 40% of potency in a sonicator (Vibra cell, Sonics & Materials Inc., Newtown, CT, USA) [38]. The whole process was carried out above the lipid’s melting point. Finally, the system was cooled to approximately 25 °C and subsequently stored at room temperature. Hybrid systems were prepared with lipid-based nanoparticles plus LAP (3%, *w/w*) by simply mixing the clay into the lipid colloidal system. All samples were homogenized in a magnetic stirrer for 48 h and then left to rest for at least five days.

#### 2.2.2. Particle Size, Polydispersity Index, and Zeta Potential

Particle size, polydispersity index (PDI), and zeta potential (ZP) were all measured by photon correlation spectroscopy (PCS), where samples were diluted (1:100) in Milli.Q water and analyzed at 25 °C with a scattering angle of 173° in particle analyzer ZetaSizer Nano ZS (Malvern^®^ Instruments, Malvern, UK). Samples with clay were diluted (5 mg·mL^−1^) in Milli.Q water and ultrasonicated to ensure complete dissolution and then analyzed under the same conditions. The particle size, polydispersity, zeta potential, and pH measurements were all performed 24 h after the preparation of all samples [39].

#### 2.2.3. High-Resolution Transmission Electron Microscopy (HRTEM, FEI TITAN G2)

All samples were diluted (1:100) in distilled water and placed on 300-mesh TEM copper grids (Neyco, Vanves, France). An aqueous uranyl acetate solution (1%, pH 4.0) was mixed with the samples to improve contrast. The excess of the staining agent was absorbed onto filter paper. Then, the grids were air-dried at room temperature and covered with a carbon film. The morphology of the uranyl-stained samples was analyzed by ultra-high-resolution transmission electron microscope and HAADF FEI TITAN G2 (UHRTEM). UHRTEM use was coupled with analytical electron microscopy performed with a SUPER-X silicon-drift windowless energy dispersive X-ray spectroscopy (EDX) detector. The AEM spectra were collected in STEM (Scanning Transmission Electron Microscopy) mode using a HAADF (High Angle Annular Dark Field) detector. X-ray chemical element maps were also collected.

#### 2.2.4. Thermal Analysis

Before performing any experiments, the samples were frozen at −20 °C for six hours and then freeze-dried for 24 h (Martin Christ Freeze-dryers, Osterode am Harz, Germany). The thermal profile of the samples was evaluated through thermogravimetry (TG) and Differential Scanning Calorimetry (DSC) after five days of the samples’ preparation. TG assays were carried out in a DTG-60 (Shimadzu^®^, Barueri, Brazil) under temperatures ranging from 25 °C to 600 °C, in a nitrogen atmosphere with a heating rate of 10 °C·min^−1^ and a flow rate of 50 mL·min^−1^. In addition, the samples, placed in aluminum sample holders, were analyzed by DSC (NETZSCH, São Paulo, Brazil) in an atmosphere of nitrogen, 50 mL·min^−1^ flow, in a temperature range of 25 °C to 150 °C, and a heating rate of 10 °C·min^−1^ [40].

#### 2.2.5. X-ray Powder Diffraction (XRD)

The diffractograms were obtained using an X-ray diffractometer (D2 Phaser–Bruker Corporation, Billerica, MA, USA), where CuKα (λ = 1.54 Å) radiation was used as the X-ray source. For the analysis, the freeze-dried samples were placed in aluminum sample supports and analyzed from 5° to 45°, with an acquisition time of 0.1 s, at 30 kV of operating voltage, and a current of 10 mA, using a Lynxeye detector.

#### 2.2.6. Rheology

The rheological behavior of the hybrid systems was investigated using a rotational rheometer (Kinexus Lab, Malvern^®^ Instruments, Malvern, UK) with cone-and-plate geometry (diameter of 20 mm, a gap between the plates of 1 mm, and an angle of 0.5 rad), and a Peltier temperature controller. Viscosity was determined by transferring approximately 1 mL of the sample to the rheometer and analyzing it at 35.5 °C (to simulate surface skin temperature) with a frequency range of 0.1–10 Hz to evaluate the stability of materials.

#### 2.2.7. Cell Assays and Fluorescence Study

Normal Human Dermal Fibroblasts were grown at 37 °C in a 5% CO_2_ and 95% air atmosphere (cell density: 20 × 10^3^/well) inside an Ibidi µ-Dish 35 mm, low for four hours, in order to obtain cell adhesion. Then, the samples with LAP (diluted 1:200, corresponding to 0.5 µg·mL^−1^ of MM or CP, in growth medium and supplemented with 1% (*v/v*) antibiotic-antimycotic solution and 10% *v/v* inactivated FBS), were put in contact with the cells for 24 h. Cell growth without a sample was used as a control. After 24 h, cell growth was terminated by withdrawing the culture medium, carefully washing three times with filtrated PBS 10% (*v/v*) (0.2-µm pore size, sterilized), and adding 50 µL 3% glutaraldehyde. Cells were fixed with glutaraldehyde for two hours at 4 °C and protected from light with aluminum foil. The glutaraldehyde was carefully withdrawn, and samples were washed three times with filtrated PBS (10% *v/v*). Subsequently, the cells were stained with the following markers added to the fixed samples in the following order: 1. Phalloidin TRICT (−20 °C) ʎ_ex_ 540–545 nm; ʎ_em_ 570–573 nm (red); 2. Hoechst 33,258 (2–8 °C) ʎ_ex_ 355 nm; ʎ_em_ 465 nm (blue). Phalloidin TRICT was chosen to stain in red the actin-cytoskeleton of fibroblasts (1:10 diluted in PBS). Phalloidin was left in contact with samples for forty minutes at room temperature and was protected from light (using aluminum foil). After the forty minutes, PBS was used to wash the sample three times. Finally, the nuclei of the cells were stained in blue with DAPI (Hoechst 332) using a dilution of 1:10,000 (in PBS) and left in contact for fifteen minutes at room temperature in the dark. Filtrated PBS 10% *v/v* was used to wash three times. In order to preserve the samples before measurement, 50 µL of additional PBS was added (to avoid dryness). They were also protected from light with aluminum foil and preserved immobile at 4 °C.

In order to evaluate the AO’s fluorescence properties, each sample was diluted (1:200) in distilled water and analyzed by spectrofluorimetry (LS50B, Perkin Elmer). The images were captured by confocal laser-scanning microscope (CLSM, Leica TCS SP8) using two channels (red and green). The 63× objective and digital zoom were employed, and the image projections (in Z dimension) were captured to verify if sample particles eventually were inside the cell cytosol.

#### 2.2.8. Statistical Analysis

Statistical differences were evaluated utilizing Student’s *t*-test, ANOVA, and Tukey post hoc tests with a significance level of 5% (*p* < 0.05). The data was calculated using Prism 6^®^ (Graphad Software, San Diego, CA, USA, 2012).

## 3. Results

### 3.1. Particle Size, Polydispersity Index, and Zeta Potential 

**Figure 1:**
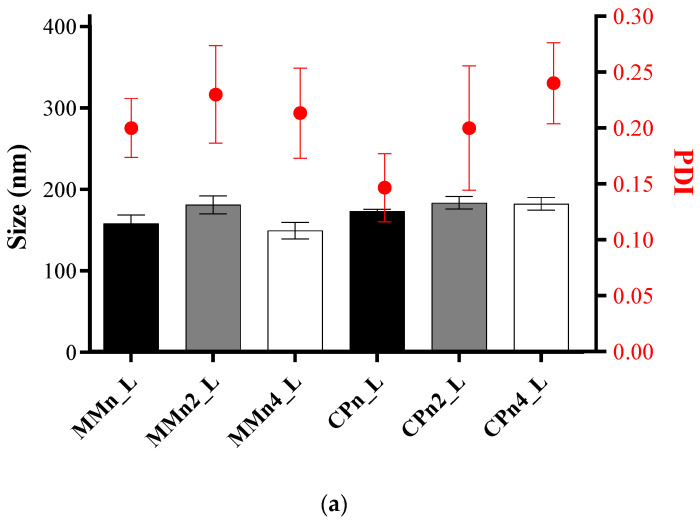

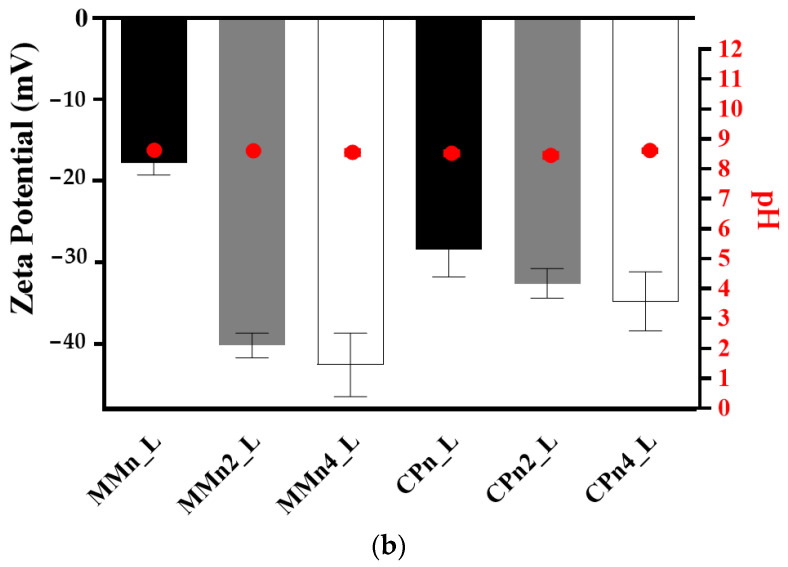
Structural properties of hybrid systems, with and without AO (0–4%, wt). DLS measurements prepared after 24 h samples are shown in terms of (**a**) size (nm), polydispersity index (PDI), and (**b**) zeta potential and pH values. Data are presented as mean ± standard deviations (*n* = 3).

Figure 1a shows the sizes and polydispersity of nanoparticles and hybrid systems around at 180 nm, and PDI > 0.3 in agreement with other studies [41]. Figure 1a shows the results of zeta potential (mV) of samples, which ranged from −16.23 ± 0.60 mV to −42.73 ± 0.29 mV. The presence of a surfactant in the formulations (PL*_bulk_*) greatly influences zeta potential values, as it contributes to the equilibrium of the repulsion forces between the particles [37]. No change in pH values was observed for samples with different liquid lipid concentrations (Figure 1b), with media values between 8.52 and 8.61 for 2 and 4% of AO, respectively. A recent study by Ferreira et al., 2021 [42] showed lower pH values for NLCs prepared with the same compounds; however, without LAP, between 5.0–6.0 for all samples. In fact, the presence of LAP provides an increase in pH due to the high pH of the raw clay. In addition, there is a tendency for carotenoid radical cations and fatty acids present in AO to provide the system’s most negative charge, as mentioned by Silva et al., 2008, Taham et al., 2015, and Kanicky et al., 2002 [43,44,45]. As observed in Figure 1b, the high concentration of AO can be responsible for forming hybrid systems with the most negative charges. The results of size, polydispersity, and zeta potential point to the presence of LAP as being an essential factor in maintaining the stability of the systems over ten weeks, mainly for formulations with MM since the samples without clay showed phase separation 48 h after their preparation, according Ferreira et al., 2021 [42]. The surface charge of the particles is one of the factors that determine physical stability. High values of zeta potential (in modulus) indicate the adequate electrostatic repulsion between the particles and better physical stability of the formulation [35,46]. LAP favored maintaining a stable system by sixty days, with a non-statistically significant variation in the ZP values (*p* > 0.5).

Bostan et al., 2021 presented satisfactory safety results for a suspension of laponite at 1.5 and 3% *w/w* in healthy volunteers. Results present laponite as a compound that does not irritate human skin, causes no trans-epidermal water loss, erythema formation, or induction of inflammatory cytokines [47]. It is worth mentioning that laponite at 1.5 and 3% have a pH of between 10 and 11.

To increase the stability of the lipid-based nanoparticles system, it is necessary to avoid sediment formation and phase separation promoted by the aging of Ostwald, beyond the flocculation, and coalescence of the particles. According to Battaglia and Gallarate, 2012 [48] and Remington, 2006 [49], the strategies to overcome these three main problems related to stability include reduction of the difference in densities between the phases (to avoid sedimentation or creaming), the use of proper colloidal stabilizers to favor electrostatic repulsion (to prevent flocculation and coalescence), and the preparation of systems with low polydispersity (to avoid the aging of Ostwald). In addition, the presence of LAP with the formation of a three-dimensional network, characterized as the ‘house of cards’, may have provided stability to lipid nanoparticles, extending its helpful pathway without significant changes in size, polydispersion, and zeta potential over the time evaluated.

### 3.2. Thermal and X-ray Diffraction Analysis

**Figure 2:**
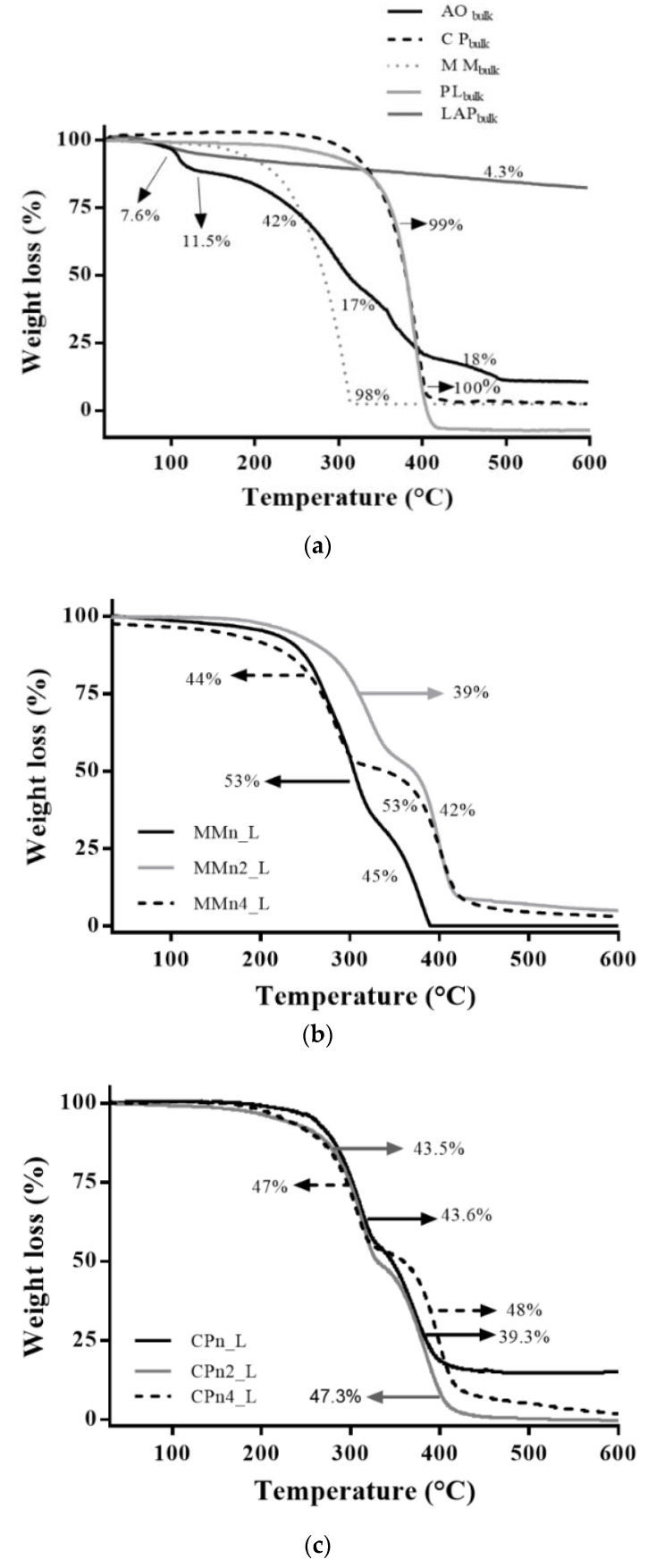
TG curves of hybrid system components AO, CP, MM, PL, and LAP (**a**), hybrid system prepared with MM (**b**) and CP (**c**).

Figure 2 shows the profile for the loss of mass during heating of the components of the systems (Figure 2a) and hybrid systems (Figure 2b,c). Figure 2a shows similar results for loss of mass of the crystalline solids (PL_bulk_, MM_bulk_, and CP_bulk_) in a single event, and these results agree with Ferreira et al., 2021 [42]. Laponite, in turn, had its mass reduced by 7.6% between 33 °C and 79 °C due to the loss of adsorption water and 4.3% slowly over the maximum heating period, due to the loss of interlamellar clay water. AO has shown a more complex profile, such as losing mass in four steps, which can be explained by the mix of substances (bixin, norbixin, and different fatty acids) present in the oil. The first step occurred between 32–101 °C with an 11.5% loss in mass due to the evaporation of the volatile components. A loss of 42.8% occurred between 103 °C and 242 °C, which can be attributed to the degradation of less stable components. According to Silva et al., 2005 and Marcolino et al., 2011, the majority of components present in AO must be stable (physically and chemically) at least up to 179 °C so they can be employed in the development of pharmaceutical dosage forms [50,51].

This is of significant importance since cis-trans isomerization of bixin occurs between 200–240 °C. The third reduction occurred between 247 °C and 323 °C, where 17.9% of the oil mass was lost due to degradation of organic compounds. The final loss was observed in the temperature range of 324 °C to 479 °C with an 18.7% loss in mass, indicating carbonization of the material. On the other hand, the nano-hybrid systems (MMn_L, MMn2_L, MMn4_L, CPn_L, CPn2_L, CPn4_L) presented good resistance to thermal variation, all initiating a loss of mass at higher temperatures. The same profile was observed in all systems, with mass loss in two consecutive events (Figure 2b,c). 

**Figure 3:**
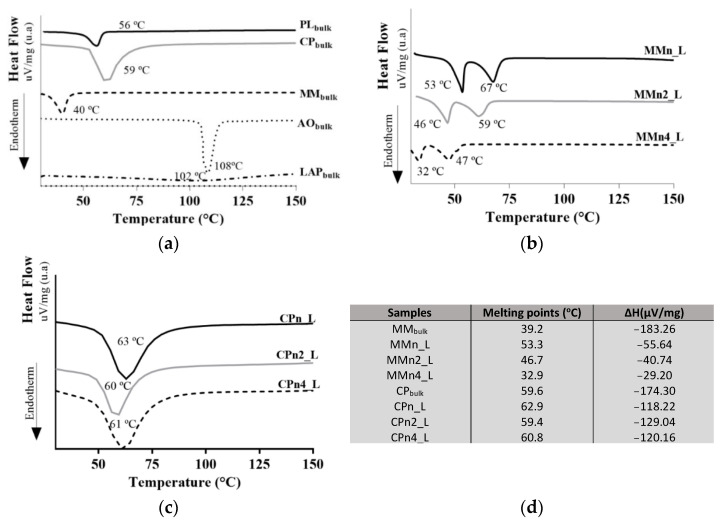
DSC curves of compounds of formulations (**a**), hybrid systems in the presence of AO at 2 and 4% (*w/w*) prepared with MM (**b**) and CP (**c**). Table (**d**) shows the melting enthalpies of the lipids and the hybrids.

Figure 3 displays the calorimetric curves of the hybrids (Figure 3b,c) and the components of the systems (Figure 3a). The colloidal stabilizer PL_bulk_ showed an endothermic peak with a melting temperature of 56 °C, in agreement with literature that refers to a melting temperature of between 55 °C and 60 °C [52], consistent with its solid crystalline nature and well-organized structure. The systems prepared with MM containing 2% and 4% AO showed differences in the system’s stability. According to Figure 2 and Figure 3, the higher the AO concentration, the greater the loss was of volatiles with a temperature ramp; when only above 300 °C, stable behavior was observed for these systems. In contrast, thermal analyses (TG and DSC) of the hybrid systems formed with CP did not show considerable changes, i.e., there was a significant variation of the melting temperature in the samples prepared with CP lipid, even with different concentrations of AO.

The MM and CP lipids had particular melting points of 39.2 °C and 59.6 °C, respectively, which corroborates with previous studies [53,54,55,56]. The LAP presented a small event at 102 °C (Supplementary Material Figure S1), which is related to the loss of adsorption water and because it is characterized as a solid material with very low crystallinity [57]. The AO peaked at a melting temperature of 108 °C, an event related to the precipitation of the bixin apocarotenoid dye, which melts at this temperature (Figure 3a) [58].

The thermograms of the hybrid systems (Figure 3b,c) showed different melting points when compared with their components (Figure 3a), as well as variations in enthalpy, suggesting the formation of new crystalline arrangements. Previous studies from our group carried out using different techniques such as DSC, XRD, and EPR helped to elucidate the arrangements of the crystalline structures, and the degree of order of the lipids present in the lipid-based nanoparticles, with and without AO. It was demonstrated that the presence of AO is one of the main factors responsible for structural modification in this lipid nanoparticle [42].

Barbosa et al., (2018) [35] showed similar results pertaining to structural information through DSC measurements on the nanoparticles prepared with the same lipids (without clay) used to carry dibucaine. The authors also demonstrated a lower crystallinity of the lipid core vs. the bulk lipid. A lower crystallinity of nanoparticles is always desirable, since this parameter is related to lower drug loss from the carrier system, providing greater stability to the formulations which increases the shelf life of the product [59,60]. The melting temperatures of most of the formulations remained above 34 °C, which is a prerequisite for maintaining the solid-state of the nanoparticles when in contact with the skin—where these formulations should be applied. Only MMn4_L formulation presented a melting point at 32 °C.

Polymorphs formed by the constituent lipids of the nanoparticles and the interaction between nanoparticles/clay can provide significant physical and chemical changes such as melting temperature of structural compounds [61,62]. In addition, these changes can affect characteristics such as spreadability, drug encapsulation, product degradation, and drug release profile. Among the steps involved in the production process of hybrid systems is the production of lipid nanoparticles. In this stage, there is recrystallization of the particles by cooling of the samples that, in general, can favor the formation of polymorphs. CP and MM lipids can undergo crystallization by forming two or three different polymorphs (α (unstable form) to β’ (intermediate form), β’ to β more ordered and stable) [63,64].

**Figure 4:**
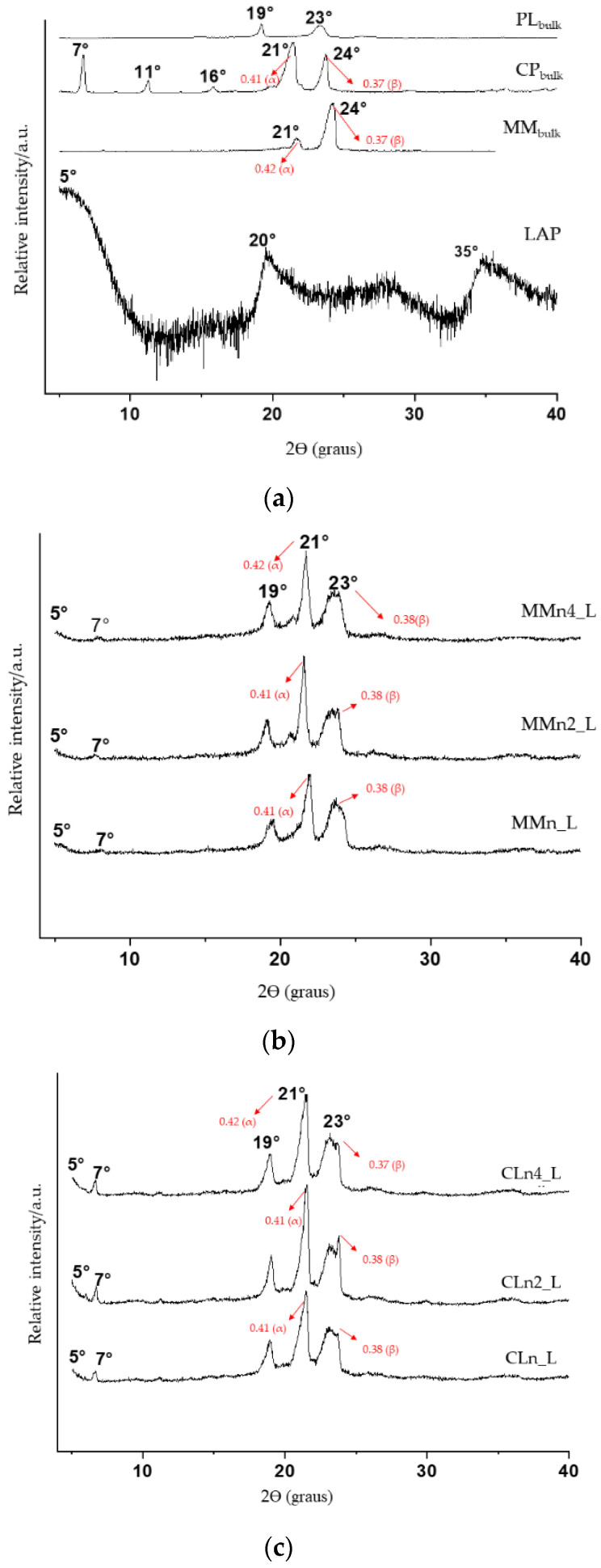
X-ray diffractograms (XRD) in terms of relative intensity vs. 2θ of isolated components (**a**), hybrid nanoparticles prepared by MM (**b**), and hybrid nanoparticles prepared by CP (**c**).

To provide information on the crystalline structure of the pure components and the hybrid systems, XRPD analyses were carried out. As shown in Figure 4a, the X-ray patterns of raw materials (MM, CP, PL, and LAP) are typical of crystalline substances. However, less intense peaks were observed only for LAP. The most intense peaks observed for PL*_bulk_* were at 19° and 23°; for CP, 7°, 11°, 16°, 21°, 24°; for MM, 21° and 24°; and LAP, 5°, 20°, and 35°.

In addition, the results showed that the long and short spacing of the crystalline lattice were modified in the hybrid systems when compared to the lipid raw materials (CP and MM), where a reduction in peak intensity was observed, and this can be related to the reduction in the degree of crystallinity of the lipid in its nanoparticulate state, resulting in a more amorphous system (Table 2). Similar results were observed by Ferreira et al., [42] without the presence of laponite. 

Figure 4b,c show changes in the intensity and position of the peaks at 21° and 24° when compared to their respective structural lipids (raw materials, Figure 4a). The same was observed in positions 19° and 23° corresponding to the PL [65,66]. These structural modifications can be explained by the amorphization of the nano-hybrid system and by the interaction between the clay and the other components of the formulation.

LAP was identified in the hybrid system due to the appearance of the band referring to the basal spacing (5°), proving the formation of a micro composite/flocculated composite with to the preservation of basal spacing (d001, calculated by the Bragg equation) in the same 2θ as in raw clay [67].

### 3.3. Rheology

LAP is a rheological modifier that is widely used in pharmaceutical or chemical products. In addition, LAP is capable of forming hydrogels organized in a ‘house of cards’ type structure with many possibilities for interactions between positively charged edges with negatively charged faces [68]. In order to obtain the formation of a hybrid lipid/clay carrier system, LAP was incorporated into the system at a concentration of 3% (*w/w*) [69]. 

**Figure 5:**
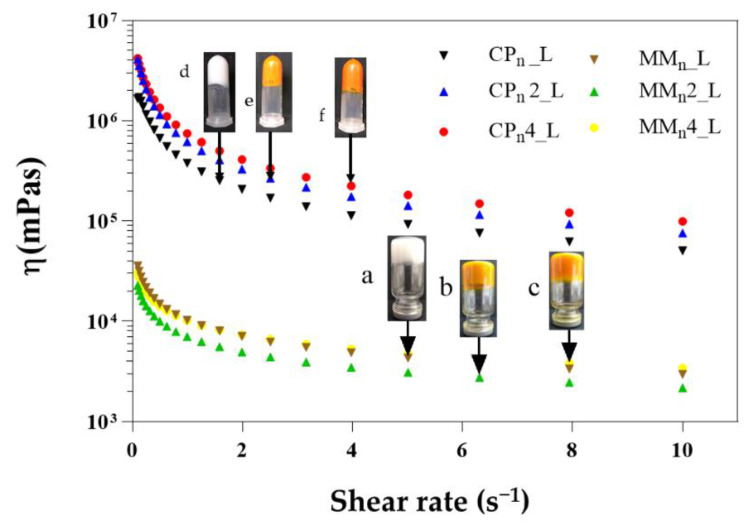
Viscosity curves of hybrid nanoparticle-imposed shear rates. Measuring time per point is 10 s. (photo of MMn_L (**a**), MMn2_L (**b**), MMn4_L (**c**), CPn_L (**d**), CPn2_L (**e**), and CPn4_L (**f**): these samples showed strong hybrid systems that can hold their weight against gravity in an inverted vial.

Figure 5 presents a viscous hybrid system with a pseudoplastic behavior, capable of holding its weight against gravity in an inverted vial (photos of hybrid systems prepared with MM; however, the same behavior was observed for samples prepared with CP), but higher viscosity for formulations with CP. A semi-solid pharmaceutical formulation is more adequate for drug delivery on the skin (dermal or topical pharmaceutical device). In addition, it can have more adhesion to the tissue, favoring the application and spreading of the active ingredient. The retention ability of topical or transdermal formulations on the skin may be reduced in products with low viscosity due to this reason; therefore, gelling agents or thickeners may be incorporated. These components can help increase viscosity and improve dermal drug delivery [70].

### 3.4. Morphology Analysis

**Figure 6:**
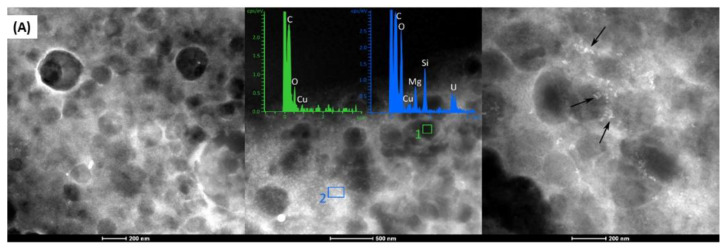

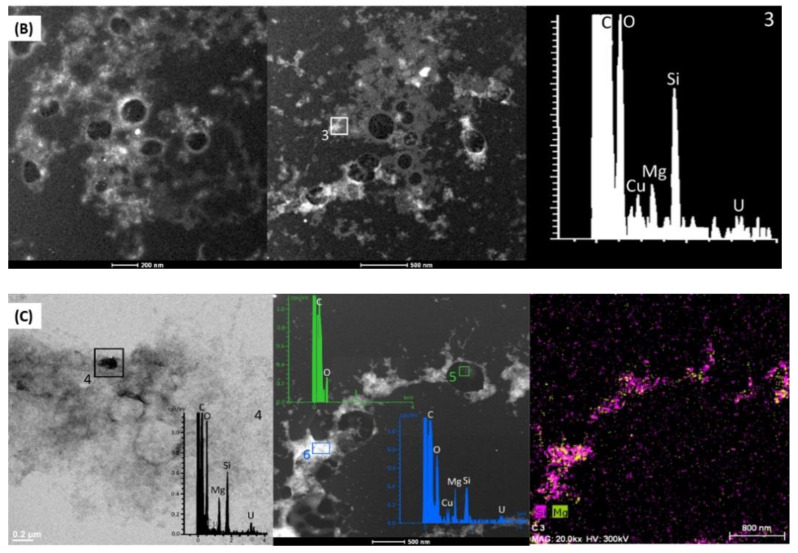
UHRTEM microphotographs, XEDS analyses, and elemental X-ray maps of MMn_L (**A**), MMn2_L (**B**), and MMn4_L (**C**). Scale bars = 200 nm.

Morphology of the hybrid systems assessed by TEM is found to be very similar: spherical structures, with a well-delimited shape and size of around 200–250 nm. Figure 6A–C show the microphotographs, the XEDS spectra, and elemental X-ray maps of the systems containing MM. The selected areas in Figure 6A represent peaks of lipids (organic components, C and O) in the green graph (region inside of nanoparticle), and clay (inorganic components, O, Mg, and Si), and 6c in the blue graph (white region, see the arrows between nanoparticles). 

A weak Cu peak corresponding to the support (grids) used to prepare the samples is also observed. Figure 6A, at the region pointed out by arrows, shows LAP outside of the nanoparticles, and no changes in shape or size. The same is observed in Figure 6B,C for MMn2_L and MMn4_L formulations, respectively. In addition, the X-ray map presented in Figure 6C highlights Si and Mg, revealing the elemental composition of LAP and its distribution in the hybrid systems. No changes were observed in the nano-hybrid prepared with CP lipids (data shown in Supplementary Material Figure S2).

### 3.5. Cell Assays

**Figure 7:**
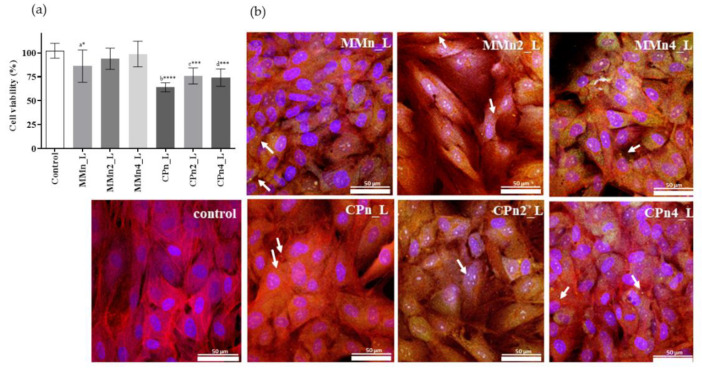
(**a**) Cell viability (%) using 3T3 in contact with nano-hybrid systems (0.5 µg mL^−1^ of lipid MM or CP) with and without AO at 2 and 4%, diluted to 0.1 and 0.2 µg mL^−1^, respectively, in comparison to the positive control GM (growth medium, as standard growth conditions) (means ± SD, *n* = 8). Statistical analyses: a*. Control vs. MMn_L, b**** = control vs. CPn_L, c*** = control vs. CPn2_L, and d*** control vs. CPn4_L, **** *p* < 0.0001, *** *p* < 0.001, * *p* < 0.05, (ANOVA and Turkey–Kramer test, 95% confidence level). (**b**) Confocal images (objective 63×); type of cell: nHDFs; Hoechst: nuclei; Phalloidin-TRIC: Actin; sample (ʎ_ex_ = 405 nm–ʎ_em_ = 460 nm); sample dilution 1:200; 48 h cell-sample contact. Abbreviations: nHDFs, normal human dermal fibroblasts.

According to Benavides and co-workers (2004), fibroblast cells (NHDF) are considered to be an important cellular model in carrying out testing for potential skin irritants [71]. Figure 7 illustrates the cytotoxic effect of nano-hybrid systems in the presence of two concentrations of AO. All samples, except CPn_L, showed a viability higher than 70% after treatment, and this suggests a complete biocompatibility in accordance to the ISO guideline ISO 10,993 part 5 [72]. In the evaluated concentrations, a higher relative toxicity was observed for the samples with CP, in relation to the control (ANOVA and Turkey–Kramer, with *p* up to <0.001), but these are however considered biocompatible considering the data dispersion. Results obtained agree with data published by Barbosa et al. in 2013 and 2018, which showed SLNs and NLCs composed of CP and MM with low toxicity in different cell types (fibroblasts and keratinocytes) [35,37].

Confocal images were obtained only after finding the best fluorescence behavior of tocotrienol molecules, determined at ʎ_ex_ = 298 and 600 nm in agreement with the literature. However, a significant fluorescence was also recorded at ʎ_ex_ = 405 nm and ʎ_em_ = 460 nm in the range of blue green which is detectable by CLSM sensitivity. Cell cytoskeleton appears in red, while cell nuclei are in blue. Moreover, sample particles adsorbed the DAPI, and in the images they appear in blue. For this reason, the same channel was used to visualize sample particles and cell nuclei contemporarily. 

Figure 7 reports the CLSM microphotographs of fibroblasts grown onto the hybrid system after three days. CLSM analyses confirm viability data for both systems (composed by MM and CP). Furthermore, the images suggest that MMn4_L provided the growth of fibroblasts with excellent attachment to the system, excellent spreadability all over the surface, and the reaching of adequate confluence, demonstrating the typical behavior of the normal stretched fibroblasts.

Regarding the presence of AO, the CLSM microphotographs also show obvious differences in performance between the hybrid systems, mainly in the high percentage of AO (4%, *w/w*) in the nano-hybrid system, confirming that AO was able to reduce the toxic effect of the hybrid system. This protection can be attributed to the antioxidative action of AO, especially by the apocarotenoids bixin and norbixin, vitamin E, and other antioxidants present in the oil [43,44,45]. This is an especially important aspect considering the use of these systems in wound healing or burn injuries. The antioxidants facilitate the healthy production of keratinocytes and fibroblasts, and tissues that will reduce scarring and skin damage. In addition, according to Packer et al., 2001, vitamin E also has skin barrier-stabilizing properties [73].

## 4. Conclusions

Nanostructured lipid carriers (NLCs) with 2 and 4% (*w/w*) of AO were obtained. However, the presence of LAP was fundamental in developing the hybrid system with thermal protection and viscosity suitable for topical application. In addition, the hybrid systems prepared with both lipids (MM and CP) demonstrated both physical and chemical stability over ten weeks. The natural compound (AO) was used due to its biological properties (antibacterial, antifungal, antileishmanial activity), its proven tissue regenerative action, and structural function in the NLCs, providing higher zeta potential values, decreased crystallinity, and higher stability to the systems. Biological evaluation performed against NHDF showed that 4% AO in samples with both lipids (MM and CP) were less cytotoxic than the other samples when compared to the control, thereby confirming that these hybrid systems loaded AO are promising candidates for topical skin application.

## Figures and Tables

**Table 1:** Composition (%, *w/w*) of hybrid nanostructured lipid carriers/clay.

Samples	Lipid (%)	AO_bulk_ (%)	PL_bulk_ (%)	H_2_O (%)	LAP (%)
MMn_L	10	-	11.7	78.3	3
MMn2_L	10	2	11.7	76.6	3
MMn4_L	10	4	11.7	74.8	3
CPn_L	10	-	11.7	78.3	3
CPn2_L	10	2	11.7	76.6	3
CPn4_L	10	4	11.7	74.8	3

**Table 2:** Main reflections and lattice spacings of the CP, MM, and hybrid nanoparticles calculated by Bragg equation. Note: α is considered the most unstable form, d values between 0.415 and 0.42 nm, β stable forms, d = 0.46 nm and β’ with 0.42 < d < 0.43 nm or 0.37 < d < 0.40 nm.

Samples	Angle 2ϴ Experimental (°)	Lattice Spacings d (nm)
MM	20.82	0.43-β’
	24.26	0.37-β’
Mn_L	21.61	0.41-α
	23.33	0.38-β’
Mn2_L	21.50	0.41-α
	23.33	0.38-β’
Mn4_L	21.33	0.42-α
	23,167	0.38-β’
CP	21.49	0.41-α
	23.74	0.37-β’
CPn_L	21.64	0.41-α
	23.42	0.38-β’
CPn2_L	21.51	0.41-α
	23.51	0.38-β’
CPn4_L	21.38	0.42-α
	23.83	0.37-β’

## Data Availability

Not applicable.

## Supplementary Materials

The following supporting information can be downloaded at: https://www.mdpi.com/article/10.3390/pharmaceutics14051067/s1, Figure S1: LAP presented a small event at 102ºC, which is related to the loss of adsorption water and because it is characterized as a solid material with very low crystallinity; Figure S2: UHRTEM microphotographs with XEDS analysis of CPn_L (a), CPn2_L (b) and CPn4_L (c). Scale bars =100–500 nm. Note: the number 1 (highlighted in green), and 2 (highlighted in red) corresponds to the region of XEDS analysis within and edge of the nanoparticle respectively.
