# Supplementary material for: Hybrid Lipid/Clay Carrier Systems Containing Annatto Oil for Topical Formulations
Source: Pharmaceutics. 2022 May 17;14(5):1067. doi: 10.3390/pharmaceutics14051067 (PMC9147908; doi:10.3390/pharmaceutics14051067)
Supplement: Supplementary file 1 [file pharmaceutics-14-01067-s001.zip › pharmaceutics-1639425-supplementary.pdf]

pharmaceutics

MDPI

Supplementary Material

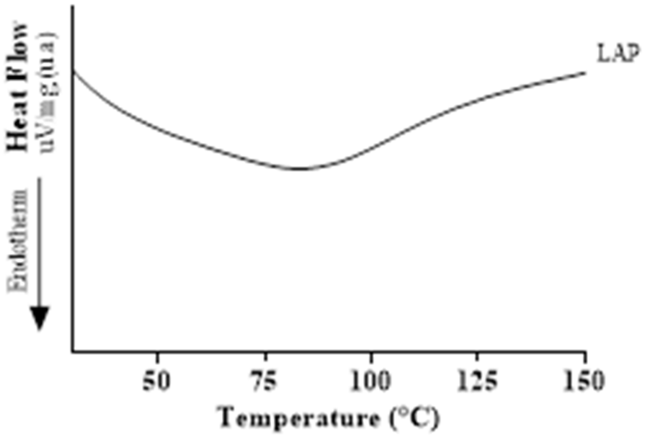

Figure S1. LAP presented a small event at 102ºC, which is related to the loss of adsorption water and because it is characterized as a solid material with very low crystallinity.

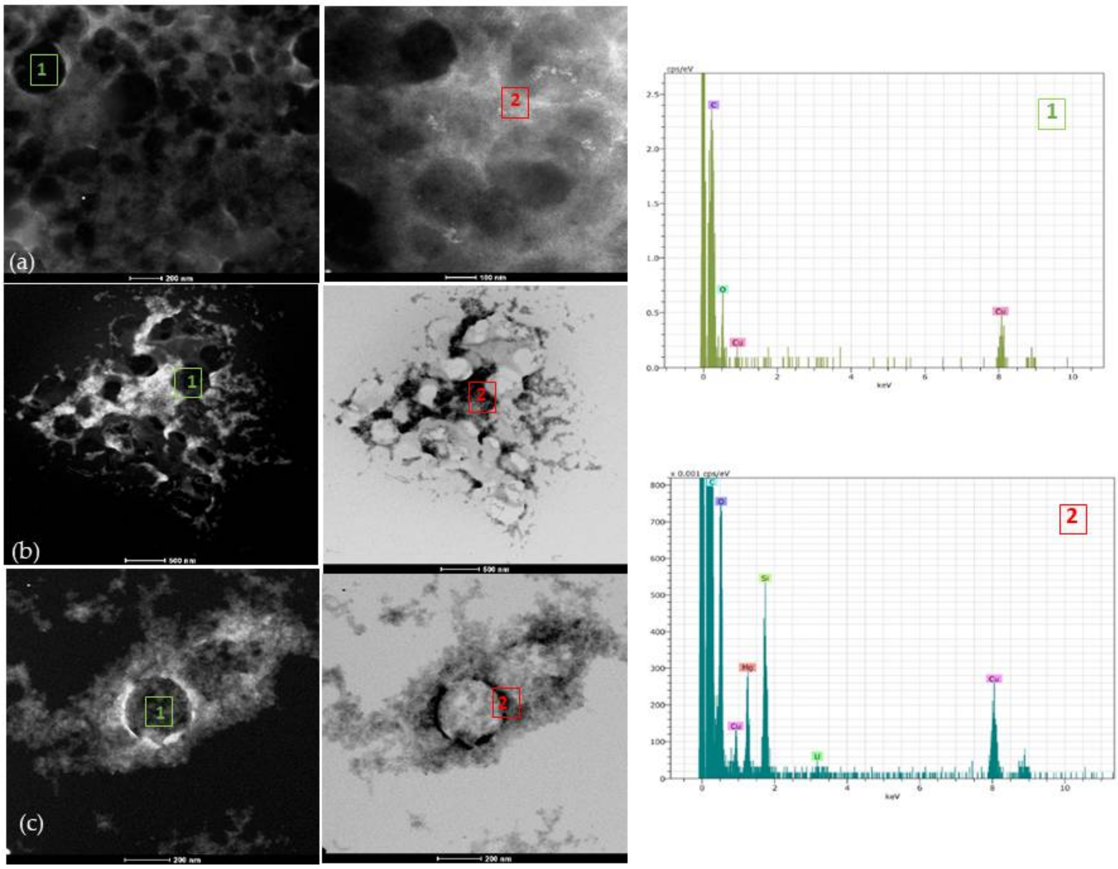

Figure S2. UHRTEM microphotographs with XEDS analysis of CPn_L (**a**), CPn2_L (**b**) and CPn4_L (**c**). Scale bars =100-500 nm. Note: the number 1 (highlighted in green), and 2 (highlighted in red) corresponds to the region of XEDS analysis within and edge of the nanoparticle respectively.
